# A new key player in VEGF-dependent angiogenesis in human hepatocellular carcinoma: dimethylarginine dimethylaminohydrolase 1

**DOI:** 10.1007/s10456-017-9567-4

**Published:** 2017-07-24

**Authors:** Nikki Buijs, J. Efraim Oosterink, Morgan Jessup, Henk Schierbeek, Donna B. Stolz, Alexander P. Houdijk, David A. Geller, Paul A. van Leeuwen

**Affiliations:** 1 Department of Surgery, University of Pittsburgh Medical Center, Pittsburgh, PA USA; 2 Department of Surgery, VU University Medical Center, PO Box 7057, 1007 MB Amsterdam, The Netherlands; 3 Department of Surgery, Medical Center Alkmaar, Trial Center Holland Health, Alkmaar, The Netherlands; 4 Department of Pediatrics, Academic Medical Center, Emma Children’s Hospital, Amsterdam, The Netherlands; 5 Department of Cell Biology and Physiology, University of Pittsburgh Medical Center, Pittsburgh, PA USA

**Keywords:** Angiogenesis, Arginine, Asymmetric dimethylarginine, Dimethylarginine dimethylaminohydrolase, Hepatocellular carcinoma, Hypoxia, Nitric oxide, VEGF

## Abstract

**Background:**

Anti-angiogenic therapies, targeting VEGF, are a promising treatment for hepatocellular carcinoma (HCC). To enhance this potential therapy, identification of novel targets in this pathway is of major interest. Nitric oxide (NO) plays a crucial role in VEGF-dependent angiogenesis. NO production depends on arginine as substrate and asymmetric dimethylarginine (ADMA) as inhibitor. Dimethylarginine dimethylaminohydrolase 1 (DDAH-1) catabolizes ADMA and therefore regulates NO and VEGF expression. This study unravels additional mechanisms to improve VEGF targeting therapies.

**Methods:**

The expression of DDAH-1 was examined in HCC specimen and non-tumorous background liver of 20 patients undergoing liver resection. Subsequently, arginine/ADMA balance, NO production, and VEGF expression were analyzed. The influence of hypoxia on DDAH-1 and angiogenesis promoting factors was evaluated in HepG2 cells and primary human hepatocytes.

**Results:**

DDAH-1 expression was significantly induced in primary HCC tumors compared to non-tumorous background liver. This was associated with an increased arginine/ADMA ratio, higher NO formation, and higher VEGF expression in human HCC compared to non-tumorous liver. Hypoxia induced DDAH-1, iNOS, and VEGF expression in a time-dependent manner in HepG2 cells.

**Conclusions:**

Our results indicate that DDAH-1 expression is increased in human HCC, which is associated with an increase in the arginine/ADMA ratio and enhanced NO formation. Hypoxia may be an initiating factor for the increase in DDAH-1 expression. DDAH-1 expression is associated with promotion of angiogenesis stimulating factor VEGF. Together, our findings for the first time identified DDAH-1 as a key player in the regulation of angiogenesis in human HCC, and by understanding this mechanism, future therapeutic strategies targeting VEGF can be improved.

## Introduction

Hepatocellular carcinoma (HCC) is a global health problem, representing the fifth most common cancer and the third most common cause of death from cancer worldwide. The incidence of this primary liver cancer has increased in the last decades [1]. Clarifying the underlying mechanisms responsible for HCC development and progression may lead to the identification of key targets for therapeutic intervention.

Hepatocarcinogenesis is a multistep process involving genetic and environmental changes, which allow the hepatocyte to escape normal control mechanisms in cell proliferation, differentiation, migration, and death, resulting in the evolvement of malignant disease [2]. Angiogenesis is a crucial process in HCC development since HCC is one of the most vascular solid tumors known. Vascular endothelial growth factor (VEGF) is the primary mediator of angiogenesis in primary liver tumors [3]. Anti-angiogenic therapies, targeting VEGF, such as sorefenib (blockage of the VEGF tyrosine kinase receptor) and bevacizumab (antibody to VEGF), have been shown to be a promising effective treatment for advanced HCC, and sorafenib has now been approved for the treatment of advanced HCC in both the USA and Europe [4, 5]. To enhance this type of targeted treatment for HCC, evaluating the effect of vertical blockade in which the VEGF pathway is interrupted at different points, is needed. This concept is appealing because it may lead to more complete blockade, block feedback loops, and have non-overlapping resistance patterns [6]. Therefore, the identification of novel targets in this pathway is of major interest.

Nitric oxide (NO), produced from arginine by the nitric oxide synthases (NOS), is a crucial molecule and regulator of angiogenesis. NO enhances the expression of other angiogenic factors, vascular permeability, perivascular cell recruitment, and vessel remodeling and maturation [7]. NO has a reciprocal role in VEGF mediated angiogenesis by stimulating the expression of VEGF and mediating its downstream angiogenic effects.

Regulation of NO production may therefore be crucial in the regulation of VEGF-dependent angiogenesis and consequently tumor progression. The amino acid arginine is the sole precursor for NO via the three isoforms of NOS: neuronal NOS (nNOS), inducible NOS (iNOS), and endothelial NOS (eNOS) [8]. Asymmetric dimethylarginine (ADMA) is a competitive inhibitor of all NOS isoforms [9]. The ratio between arginine and ADMA is the indicator for NOS activity and therefore NO formation. ADMA is predominantly catalyzed by the enzyme dimethylarginine dimethylaminohydrolase (DDAH) [10]. Thus, ADMA and DDAH have an important regulatory role in NO production, and the balance between arginine and ADMA (arginine/ADMA ratio) is the preserving factor in this pathway [7, 11]. The DDAH enzyme has two isoforms: DDAH-1 is the most important isoform in ADMA metabolism, and the liver is a major expression site. DDAH-2 is particularly expressed in vascular tissue, and it was found that ADMA levels do not evidently depend on DDAH-2 activity [12].

The role of ADMA and the DDAH isoforms in angiogenesis is excessively studied in cardiovascular settings. It was shown that ADMA and DDAH-1 metabolism significantly influences NO formation and hereby plays a role in the regulation of endothelial function, vascular condition and development [13–15]. Moreover, increased DDAH-1 expression is associated with upregulation of angiogenic factors, most importantly VEGF [16]. These findings suggest that DDAH-1 and ADMA may be potential anti-angiogenic targets in cancer treatment. Insights in the mechanisms of action of these players in HCC development may provide new pointers in treatment and amplify the battle against this malignancy.

We hypothesized that DDAH-1 may be a potential target in HCC therapy. Therefore, we evaluated whether DDAH-1 expression is increased in HCC tissue compared to non-cancerous liver tissue of 20 patients who underwent hepatic resection. In the same tissue samples, we determined the arginine/ADMA ratio, NO formation, and VEGF expression. In addition, we determined the effect of hypoxia on DDAH-1, iNOS, and VEGF expression in HCC cells.

## Materials and methods

### In vivo materials

Tissue and serum samples were collected from 20 HCC patients undergoing surgical resection at the Liver Cancer Center of the University of Pittsburgh Medical Center under an institutional review board (IRB)-approved protocol. From each patient, a HCC sample, a non-tumorous liver sample, and serum were collected. Serum of these patients undergoing hepatic resection for HCC (*n* = 20) and from a control group of patients undergoing hepatic resection for benign lesions (*n* = 10) was collected preoperatively. Patient tissue samples and serums were stored at −80 °C until analysis. Informed consent was obtained from all individual participants included in the study.

### In vitro materials

Experiments were also done in cell cultures. The HepG2 cell line (HB-8065) was purchased from and tested by American Type Culture Collection (Manassas, VA), and the primary hepatocytes were obtained from histologically normal liver under an IRB-approved protocol.

### Immunoblotting analysis

Whole-cell protein was extracted from the HCC and corresponding non-tumorous tissue samples with tissue protein extraction reagent (Pierce Biotechnology, Rockford, IL). Whole-cell protein of the cultured primary hepatocytes and HepG2 cells were extracted with cell lysis reagent (Sigma, St. Louis, MO). Samples were quantified, and 20–100μg of total protein was separated by gel electrophoresis. The separated proteins were transferred to a membrane, and membranes were blocked in 5% low-fat milk, and western blotting was performed with antibodies against DDAH-1 (Santa Cruz Biotechnology Inc., Dallas, TX) (1:500), iNOS (BD Biosciences, San Jose, CA) (1:500), VEGF (Santa Cruz Biotechnology Inc., Dallas, TX) (1:500), and β-actin (Abcam, Cambridge, MA) (1:1000) diluted in 1% low-fat milk solutions over night. The membranes were washed with Tris-buffered saline with Tween (TBST) and incubated with horseradish peroxidase-conjugated secondary antibodies and washed again with TBST. Chemiluminescent substrate (Thermo Fisher Scientific, Rockford, IL) was added to the membranes for 3–7 min. Excessive substrate was removed, and the membranes were placed in transparent plastic sheets and exposed to film (Laboratory Product Sales Inc, Rochester, NY).

### Immunofluorescence

HCC tissue samples and non-tumorous liver tissue samples were fixed in 2% paraformaldehyde for 2 h and placed in 30% sucrose for 24 h. The fixed tissue samples were placed in liquid nitrogen cooled 2-methylbutane, and cryostat sections (6–10 μm) were placed onto glass slides. After rehydration with phosphate-buffered saline, the tissue sections were permeabilized with 0.1% Triton X-100 (Sigma, St. Louis, MO) and washed in protein blocking buffer (PBB; 0.5% bovine serum albumin in phosphate-buffered saline) after each treatment. Tissue sections were blocked in the serum of the host of the secondary antibody for 45 min at room temperature (Sigma, St. Louis, MO; Millipore, Billerica, MA). After 1-h incubation with specific primary antibody for DDAH-1(Santa Cruz Biotechnology Inc., Dallas, TX) (1:100 dilution in PBB with 0.1% triton X-100), iNOS (BD Biosciences, San Jose, CA) (1:100 dilution in PBB), and VEGF (Abcam, Cambridge, MA) (1:250 dilution in PBB) at room temperature, tissue sections were treated with species specific secondary antibody and fluor-conjugated phalloidin (Invitrogen, Carlsbad, CA) for 1 h at room temperature. Hoechst stain was added for 30 s. Fluorescent images were captured with a confocal microscope (FluoView 1000, Olympus, Tokyo, Japan). Quantification analyses were performed by means of traditional binary threshold using the negative staining control as a reference point for staining intensity of an area per nucleus.

### Arginine and ADMA measurements

The arginine/ADMA ratio was determined by measuring arginine and ADMA concentrations by using a previously described liquid chromatography mass spectrometry method [17].

### Serum and tissue NO assay

NO in serum and tissue was predicted by measuring nitric oxide metabolites (NOx) by the Griess reaction after conversion of nitrate to nitrite using a nitric oxide colorimetric assay kit (Cayman Chemical Company, Ann Arbor, MI). The NOx measurements were performed in preoperative serum samples of the HCC patients (*n* = 20); the serum of patients with benign liver lesions functioned as control (*n* = 10). Also, tissue homogenates of 20 paired HCC samples, and their non-tumor counterparts were analyzed. Serum and tissue samples preparation was according to the manufacturer’s protocol. NOx concentrations were defined as μM per μg protein in tissue homogenates and as μM per L serum.

### Hypoxia experiment

HepG2 cells were seeded on 12 sterile culture dishes. After 24 h of incubation, the cells were washed and incubated under normoxic (21.0% O_2_) and hypoxic (1.0% O_2_) conditions in duplicate for 0, 1, 3, 6, 12, and 24 h. DDAH-1, iNOS, and VEGF expressions were determined by immunoblotting as described above.

### Statistical analysis

Results are expressed as mean ± standard error of the mean (SEM). Statistical analysis was performed using the Student’s *t* test. SPSS 20.0 for Windows (SPSS Inc., Chicago, IL) was used for statistical analysis. *P* < 0.05 was considered as statistically significant.

## Results

**Fig. 1:**
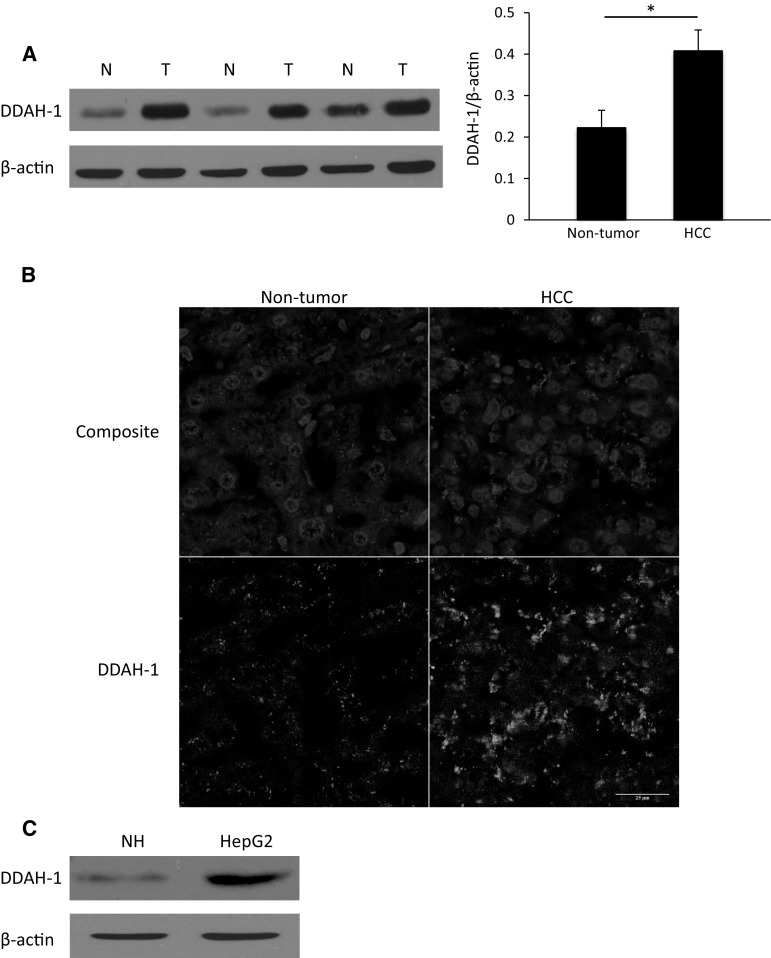
Overexpression of DDAH-1 in HCC. **a** DDAH-1 protein levels were measured with immunoblotting in paired HCC samples and their non-tumorous counterparts. Protein expression results were normalized to internal control β-actin. *P < 0.05. *N* non-tumorous liver (*n* = 20), *T* HCC tumor (*n* = 20). Imagings shown are representative results of three patients. **b** Non-tumorous human liver and human HCC tissues were stained for DDAH-1. In the composite images: *Red*, DDAH-1; *blue*, nuclei. In the single DDAH-1 channel images: DDAH-1, *gray*. **c** Expression of DDAH-1 in in vitro cultured human primary hepatocytes and in a HCC cell line was detected with immunoblotting analysis. (Color figure online)

Patient characteristics are shown in Table 1. We found a higher expression of DDAH-1 in the HCC tumor samples compared to the corresponding non-tumorous liver tissue samples (Fig. 1a). Immunofluorescence confirmed increased DDAH-1 expression in the primary HCC tumors compared to non-tumorous liver tissue. DDAH-1 was localized in hepatocytes and most abundant in HCC cells, whereas expression of DDAH-1 in endothelial cells of vascular structures was not observed (Fig. 1b). In vitro experiments also showed that the expression of DDAH-1 was increased in the HCC cell line HepG2 compared to normal primary hepatocytes (Fig. 1c).

**Table 1:** Patient characteristics

Clinicopathological features	Value
Number of patients	20
Sex	8 female; 12 male
Age (years)	66 ± 3
Cirrhosis	5 (25%)
Hepatitis	
HBV	1 (5%)
HCV	5 (25%)
Non-viral hepatitis	
ETOH	2 (10%)
NASH	8 (40%)
Other	1 (5%)
Mean tumor size (cm)	6.8 ± 1
No. of tumor nodules	
1	10 (50%)
≥2	10 (50%)
Differentiation	
Well	4 (20%)
Moderate	16 (80%)
Poor	0 (0%)
Vascular invasion	11 (55%)
Stage	
I	5 (25%)
II	15 (75%)
III	5 (25%)

*HBV* hepatitis B virus, *HCV* hepatitis C virus, *ETOH* alcohol-induced hepatitis, *NASH* non-alcoholic steatohepatitis

**Fig. 2:**
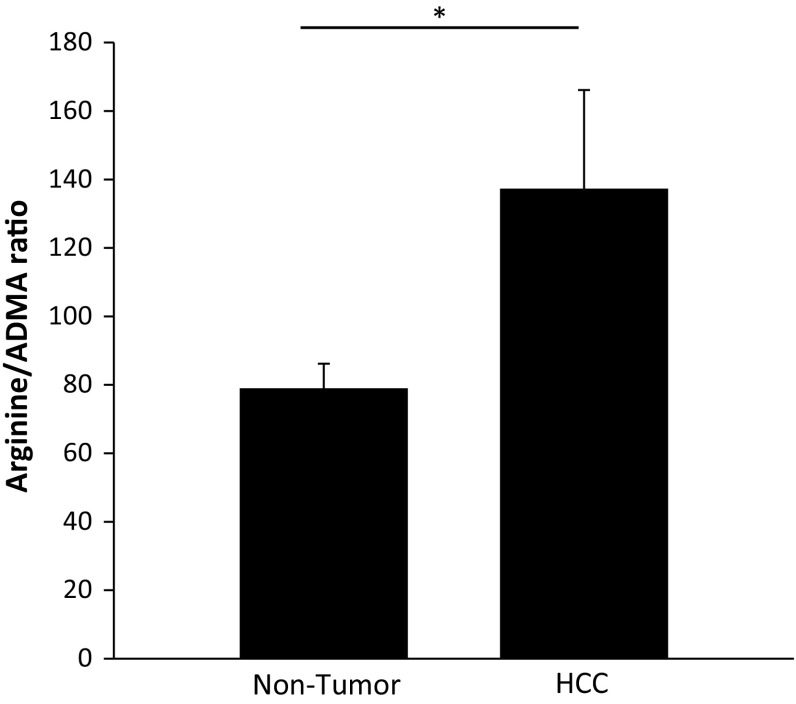
Increased arginine/ADMA ratio in HCC tissue. Arginine and ADMA concentrations in HCC tissue (*n* = 20) and non-tumorous liver tissue (*n* = 20) were measured with a liquid chromatography mass spectrometry method. *P < 0.05

To determine the effect of the observed increase in DDAH-1 expression on the arginine/ADMA ratio (the indicator for NO production), arginine and ADMA concentrations in HCC and the paired non-tumorous tissue of 20 patients were measured using mass spectrometry. A higher arginine/ADMA ratio is linked to a higher NO production. The arginine/ADMA ratio was 74% higher in HCC tissue compared to the non-tumorous liver tissue (137 ± 29 vs. 79 ± 7, respectively) (Fig. 2).

**Fig. 3:**
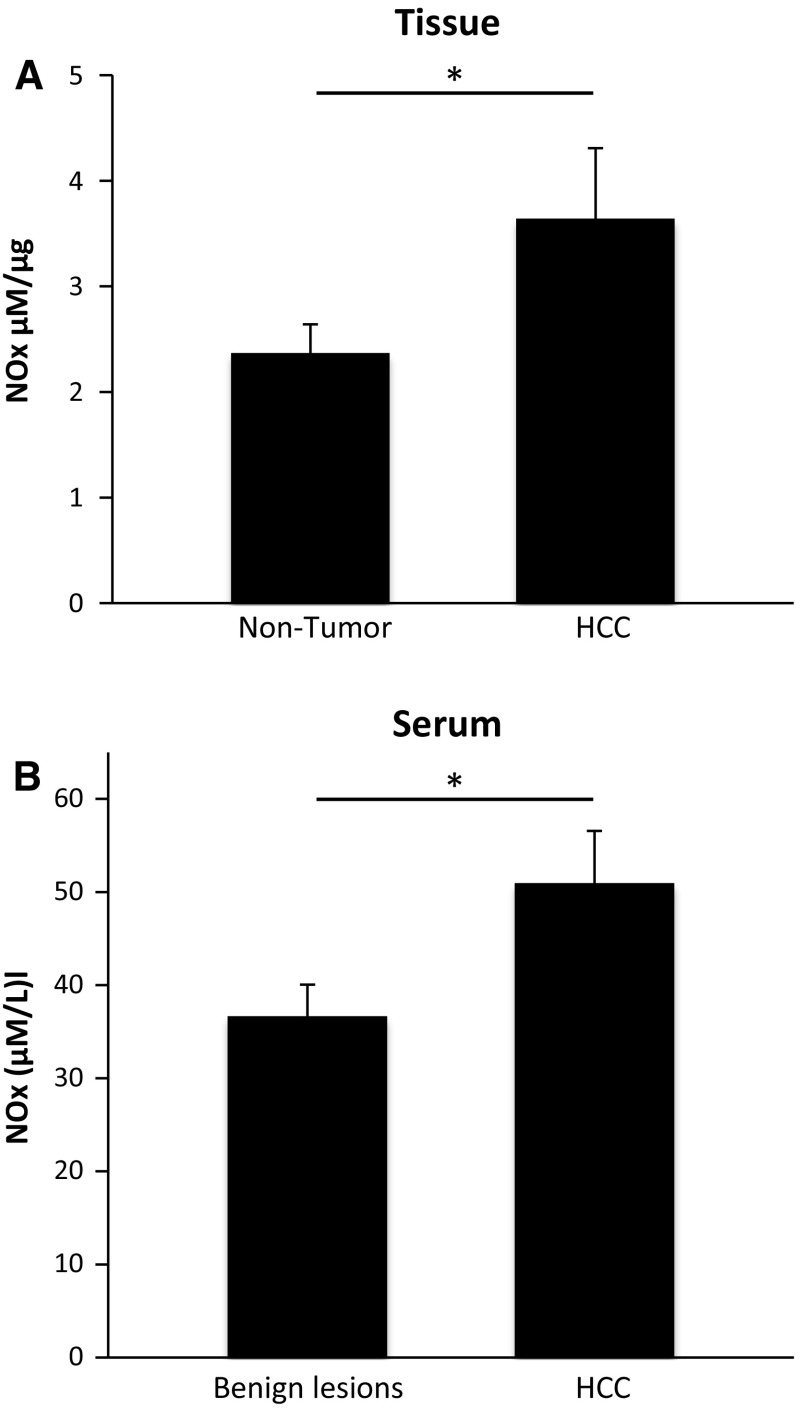
NO metabolites were increased in HCC tissue (*n* = 20) compared to non-tumorous liver tissue (*n* = 20) and were higher in serum of HCC patients (*n* = 20) compared to serum of patients with benign lesions (*n* = 10). NO metabolites were analyzed in tissue (**a**) and serum (**b**) by using a Griess reagent protocol. NOx concentration was defined as μM per μg protein in tissue homogenates and as μM per L serum. *P < 0.05

To study the effect of the increased DDAH-1 expression and subsequently increased arginine/ADMA ratio in HCC on NO formation in these patients, NO metabolites were measured in the same tissue homogenates. We found significant higher NOx levels in the HCC homogenates compared to the non-tumorous liver homogenates (Fig. 3a). Furthermore, NOx levels in serum of HCC patients were significantly higher compared to non-cancer patients (Fig. 3b).

**Fig. 4:**
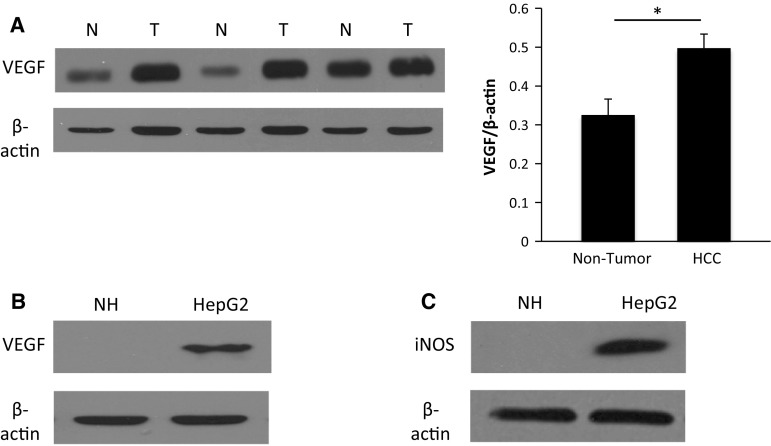
Overexpression of angiogenesis promoting factors VEGF and iNOS in HCC. **a** VEGF protein levels were measured with immunoblotting analysis in paired HCC samples (*n* = 20) and their non-tumorous counterparts (*n* = 20). Protein expression results were normalized to internal control β-actin. *P < 0.05. *N* non-tumorous liver, *T* tumor. Expression of VEGF (**b**) and iNOS (**c**) in in vitro human primary hepatocytes and in a HCC cell line were detected with immunoblotting analysis

We also found that the increased DDAH-1 expression in HCC tissue was accompanied by an increased expression of the angiogenesis stimulating factor VEGF (Fig. 4a). In vitro, the HCC cell line also showed an increased VEGF and iNOS expression compared to the primary hepatocytes (Fig. 4b, c).

**Fig. 5:**
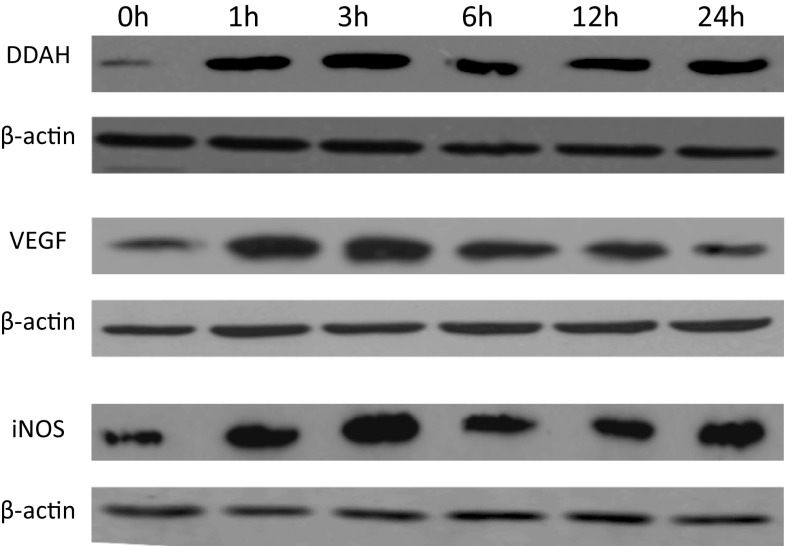
Hypoxia induces overexpression of DDAH-1, VEGF and iNOS in HCC cells in a time-dependent manner. Expression of DDAH-1, VEGF and iNOS was determined by immunoblotting in HepG2 cells subjected to a time course up to 24 h of hypoxia (1% O_2_)

Hypoxia is often seen in solid tumors, including primary liver tumors. This is confirmed in a recent study by our group, showing that hypoxia-inducible factor-1 alpha (HIF-1α) is indeed substantially increased in HCC specimens, compared to the non-tumorous specimens of our patients [18]. Hypoxia and the subsequent expression of HIF-1α have shown to induce the expression of iNOS and VEGF [19, 20]. To determine whether tumor hypoxia also influences the expression of DDAH-1, we performed this in vitro experiment: HepG2 cells were cultured under normoxic and hypoxic conditions. DDAH-1 expression increased in hypoxia in a time-dependent manner. The expression of the angiogenesis promoting factors iNOS and VEGF showed a similar increase in time in the hypoxic HepG2 cells (Fig. 5).

## Discussion

Despite advances in surgical and ablative techniques in the past decades, HCC is still a leading cause of cancer-related death worldwide. Many patients are diagnosed with HCC in an advanced state of malignant disease, and there remains a lack of effective chemotherapeutic treatment for widely progressive disease [21, 22]. Recently the therapeutic application of anti-angiogenetic agents in HCC, targeting the VEGF pathway, has shown promising results in the treatment of advanced HCC. The search for novel targets in the VEGF pathway in HCC to complement this therapy may clarify molecular and metabolic changes in hepatocarcinogenesis and improve therapeutic effects. Therefore, obtaining more insight in these mechanisms of action is an important objective in advance of novel strategies to prevent and treat primary liver tumors.

With regard to our results, we hypothesize on the HCC-induced mechanisms to allow malignant outgrowth: Solid tumors create an environment which prevents the immune system from counteracting tumor development and promote malignant progression, indicating tumor sprouting and angiogenesis. NO augments DNA synthesis, cell proliferation, and migration and mediate the function of multiple angiogenetic factors, such as VEGF, and hence NO is essential for tumor progression. HCC cells stimulate the formation of iNOS derived NO by the expression of cytokines, e.g., tumor necrosis factor alpha (TNF-α) and HIF-1α. The arginine/ADMA ratio is the preserving factor in NO synthesis, since arginine is its sole precursor and ADMA is the competitive antagonist. DDAH catabolizes ADMA, and therefore, enhanced DDAH activity results in an increase in NO formation. Enhanced DDAH expression could be a mediating step in the pathway of NO synthesis induced by tumor cytokine activation to promote tumor growth. Our study shows a clear association between DDAH-1 expression, arginine/ADMA ratio, and subsequently NO formation and VEGF expression in human HCC.

One of the most prominent factors implicated in angiogenesis and tumor progression is VEGF. This angiogenetic mediator induces vascular sprouting, increases endothelial permeability, and maintains vascular integrity in the tumor [23]. NO derived from tumor-induced iNOS expression is also a key regulator of angiogenesis and tumor growth. This inducible isoform of NOS is only expressed in stressed tissue. Whereas nNOS and eNOS produce small amounts of NO in a pulsatile manner, iNOS continuously produces high amounts of NO [24]. NO induces the expression of VEGF and mediates its angiogenesis stimulating effects. VEGF on its turn stimulates the expression of iNOS and its continuous production of NO. Furthermore, the expressions of iNOS and VEGF are closely related to tumor angiogenesis and are involved in tumor metastasis and invasiveness [25, 26].

Arginine is used by all NOS isoforms to form NO. ADMA regulates this NO production by competing with arginine for NOS and therefore blocking the formation of NO. NOS is mainly localized in the cell, and thus the intracellular ADMA and arginine levels influence NO production. Extracellular ADMA is also an antagonist to extracellular arginine on cell membrane transporter level, whereas they are both transported into the cell via cationic amino acid transporters of system y+ [27, 28]. Thus, NO production depends on the arginine/ADMA ratio, which was also shown in our results.

ADMA is catabolized by DDAH and the high DDAH-1 expression as found in our human HCC specimens was associated with an increase in the arginine/ADMA ratio and higher NO formation. Although the role of both arginine and NO are excessively studied in the oncological setting, studies on the role of ADMA and DDAH in human tumor development and progression are lacking. Though, ADMA and DDAH are widely analyzed in the cardiovascular setting as regulators of NO dependent endothelial function, vascular tone, organ perfusion, and vascular proliferation [16]. Low DDAH activity and subsequently increased ADMA levels cause a deficiency in NO bioavailability, and this results in cardiovascular dysfunction. There are only a few studies that translated the role of ADMA and DDAH in the oncological setting. Those studies reported that both isoforms of DDAH indeed may play a role in the development of tumor vasculature [29].

A key role for DDAH in tumor angiogenesis is supported by studies showing that DDAH overexpression activates angiogenic pathways in vitro. Vanella et al. [30] showed that DDAH-2, iNOS, and VEGF expressions were higher in a prostate cancer cell line compared to cells that represent benign prostate hypertrophy. Consistent to our results, Kostourou et al. [31] showed in in vivo experiments in rats bearing glioma xenografts that overexpression of DDAH-1 and a subsequent decreased inhibiting effect of ADMA on NOS result in increased tumor growth, tumor vascularization, and VEGF secretion. We now report that DDAH-1 is overexpressed in human HCC compared to non-tumorous liver, that this increase in DDAH-1 expression results in enhanced NO formation and is associated with stimulation of angiogenetic factors. More studies are needed to further unravel the mechanisms behind this pathway; for example, DDAH-1 knockdown experiments in rodents may gain more insight into the point of action of DDAH-1 in VEGF-dependent angiogenesis and tumor growth of HCC.

HCC shows signs of hypoxia, which is associated with tumor progression and a poor prognosis. Increased expression of angiogenesis promoting factors is required for tumor growth, counteracting cancer cell hypoxia [32]. However, the mechanism by which deprivation of adequate oxygen supply influences cancer progression is still unclear. Previous studies showed that NO production is primarily present in tumor areas between viable and necrotic tumor regions, the hypoxic area [33]. Hypoxic tissue induces the expression of hypoxia-inducible factor-1 alpha (HIF-1α), which stimulates the expression of iNOS and VEGF, which was also seen in our hypoxia experiment in the HCC cells. In the clinical setting, malignancies with high iNOS and VEGF expression typically present as highly vascularized tumors [34]. When inhibiting iNOS and VEGF activity in tumors, the tumor vasculature becomes dysfunctional and tumor perfusion is not effective, resulting in decreased tumor growth [35, 36]. Our study also aimed to determine the effect of tumor hypoxia on DDAH-1 expression and the arginine/NO pathway. We found that hypoxia also induces DDAH-1 expression in HCC cells.

Our results suggest that DDAH has a regulating role in tumor angiogenesis in human by stimulating NO formation and VEGF expression. Multiple trials already studied the clinical effects of angiogenesis targeted therapy [37, 38]. Incomplete knowledge and complexity of the underlying mechanisms of the hepatocarcinogenesis are most likely to be the restrictive factors in refining these therapies [39]. The results of our study further elucidate the complex tumor-promoting pathways in HCC. Whereas VEGF inhibitors alone show promising effects in the treatment of HCC, our study suggests an additional novel role for DDAH-targeted therapy in HCC to significantly improve clinical outcome in HCC patients. As stated above, tumors with high DDAH-1 expression grow almost twice as fast as controls, which clearly shows the importance of DDAH in tumor progression [31]. Combined with our results, inhibiting DDAH may serve as a potential antitumor strategy. Therefore, we hypothesize the development of multiple-targeted inhibitors to control NO formation and subsequently angiogenesis and tumor progression.

In conclusion, our data show that DDAH-1 is overexpressed in HCC and plays a pivotal role in the regulation of hypoxia-induced angiogenesis by regulating the NO/VEGF pathway. These findings suggest that DDAH-1 may serve as a promising target for development of novel therapeutic agents for HCC and that future studies are needed to further explore the role of DDAH-1 in the hepatocarcinogenesis and as a targeted agent in HCC therapy development.
